# Pdcd4 restrains the self-renewal and white-to-beige transdifferentiation of adipose-derived stem cells

**DOI:** 10.1038/cddis.2016.75

**Published:** 2016-03-31

**Authors:** Y Bai, Q Shang, H Zhao, Z Pan, C Guo, L Zhang, Q Wang

**Affiliations:** 1 Department of Immunology, Shandong University School of Medicine, Jinan, Shandong 250012, China

## Abstract

The stemness maintenance of adipose-derived stem cells (ADSCs) is important for adipose homeostasis and energy balance. Programmed cell death 4 (Pdcd4) has been demonstrated to be involved in the development of obesity, but its possible roles in ADSC function and adipogenic capacity remain unclear. In this study, we demonstrate that Pdcd4 is a key controller that limits the self-renewal and white-to-beige transdifferentiation of ADSCs. Pdcd4 deficiency in mice caused stemness enhancement of ADSCs as evidenced by increased expression of CD105, CD90, Nanog and Oct4 on ADSCs, together with enhanced *in situ* proliferation in adipose tissues. Pdcd4 deficiency promoted proliferation, colony formation of ADSCs and drove more ADSCs entering the S phase accompanied by AKT activation and cyclinD1 upregulation. Blockade of AKT signaling in Pdcd4-deficient ADSCs led to a marked decline in cyclinD1, S-phase entry and cell proliferation, revealing AKT as a target for repressing ADSC self-renewal by Pdcd4. Intriguingly, depletion of Pdcd4 promoted the transdifferentiation of ADSCs into beige adipocytes. A reduction in lipid contents and expression levels of white adipocyte markers including C/EBP*α*, PPAR-*γ*, adiponectin and *α*P2 was detected in Pdcd4-deficient ADSCs during white adipogenic differentiation, substituted by typical beige adipocyte characteristics including small, multilocular lipid droplets and UCP1 expression. More lactate produced by Pdcd4-deficient ADSCs might be an important contributor to the expression of UCP1 and white-to-beige transdifferentiation. In addition, an elevation of UCP1 expression was confirmed in white adipose tissues from Pdcd4-deficient mice upon high-fat diet, which displayed increased energy expenditure and resistance to obesity as compared with wild-type obese mice. These findings provide evidences that Pdcd4 produces unfavorable influences on ADSC stemness, which contribute to adipose dysfunction, obesity and metabolic syndromes, thereby proposing Pdcd4 as a potential intervening target for regulating ADSC function.

Adipose-derived stem cells (ADSCs) are mesenchymal stem cells derived from various types of adipose tissues including visceral and subcutaneous fat.^1^ ADSCs express mesenchymal stem cell-related markers CD105, CD90, CD29, CD44 and CD73, and possess stemness properties such as self-renewal capacity and multipotency.^2, 3, 4, 5^ The stemness of adult stem cells is essential for tissue homeostasis and pluripotency,^6, 7, 8^ which is also applied to ADSCs and adipose homeostasis. ADSCs are the main source of adipocytes in adipose tissues, which have an essential role in energy balance and nutrition homeostasis. ADSCs can differentiate into most mesenchymal cell types including adipocytes, chondrocytes and osteoblast *in vitro*.^3, 9^ *In vivo*, ADSCs contribute to *de novo* adipogenesis and adipocyte hyperplasia in response to high-fat diet (HFD) challenge.^10^ In human subjects and animal models with obesity, the shortage of ADSCs impedes adipogenesis and drives adipocyte hypertrophy, thus leading to adipose dysfunction and further metabolic syndromes.^10, 11, 12^ Therefore, the capacity of ADSCs for effective proliferation and differentiation has pivotal roles in the physiological function of adipose tissues.

Two different types of adipose tissues, white adipose tissues (WAT) and brown adipose tissues (BAT), reside in mammals. WAT is responsible for fat storage and energy balance. Excessive energy causes adipocyte hypertrophy and pathological expansion of WAT especially visceral WAT, which contributes to WAT inflammation, insulin resistance and subsequent metabolic syndromes. In contrast, BAT is specialized to burn fat for thermogenesis through uncoupled respiration in response to cold or excess feeding.^13, 14, 15^ Recently, a newly identified type of brown-like fat cells, termed beige or brite adipocytes, were found within certain WAT depots upon appropriate stimulation. Beige adipocytes are capable of expressing BAT-specific uncoupling protein 1 (UCP1) and gene expression pattern that is distinct from either white or classical brown adipocytes.^10, 16, 17^ As a protein pumping proton into the mitochondrial matrix, UCP1 uncouples oxygen consumption from ATP synthesis, thereby triggering a program of respiration and energy expenditure.^18, 19, 20^ Accumulating evidences have demonstrated the occurrence of UCP1-positive beige fats in WAT, also called browning of WAT, produces beneficial effects against obesity, diabetes and metabolic diseases.^15, 21, 22, 23, 24^ As the differentiation of ADSCs into white or beige adipocytes may determine the development of obesity or not, genes or proteins that control the differentiation destination of ADSCs would become promising targets for obesity treatment.

Programmed cell death 4 (Pdcd4) was initially found to be upregulated during apoptosis.^25, 26, 27^ So far, Pdcd4 has been well recognized as a tumor suppressor and regulates gene expression through influencing translation and transcription.^28, 29, 30^ Recently, emerging evidences have demonstrated the involvement of Pdcd4 in inflammatory or metabolic diseases.^31, 32, 33^ We have previously shown that in response to HFD, Pdcd4 promotes the pathological expansion of epididymal WAT and the development of obesity partially through inhibiting a modulator of lipid homeostasis, LXR-*α*.^32^ Considering the critical roles of ADSCs in adipose homeostasis, the possible effects of Pdcd4 on ADSCs remain to be clarified. In the present study, we provide evidences that Pdcd4 acts as a controller that limits ADSC self-renewal and white-to-beige transdifferentiation, which could be another contributor to adipose dysfunction and obesity.

## Results

### Pdcd4 deficiency increases the expression levels of stem cell-related phenotypes on ADSCs

**Figure 1:**
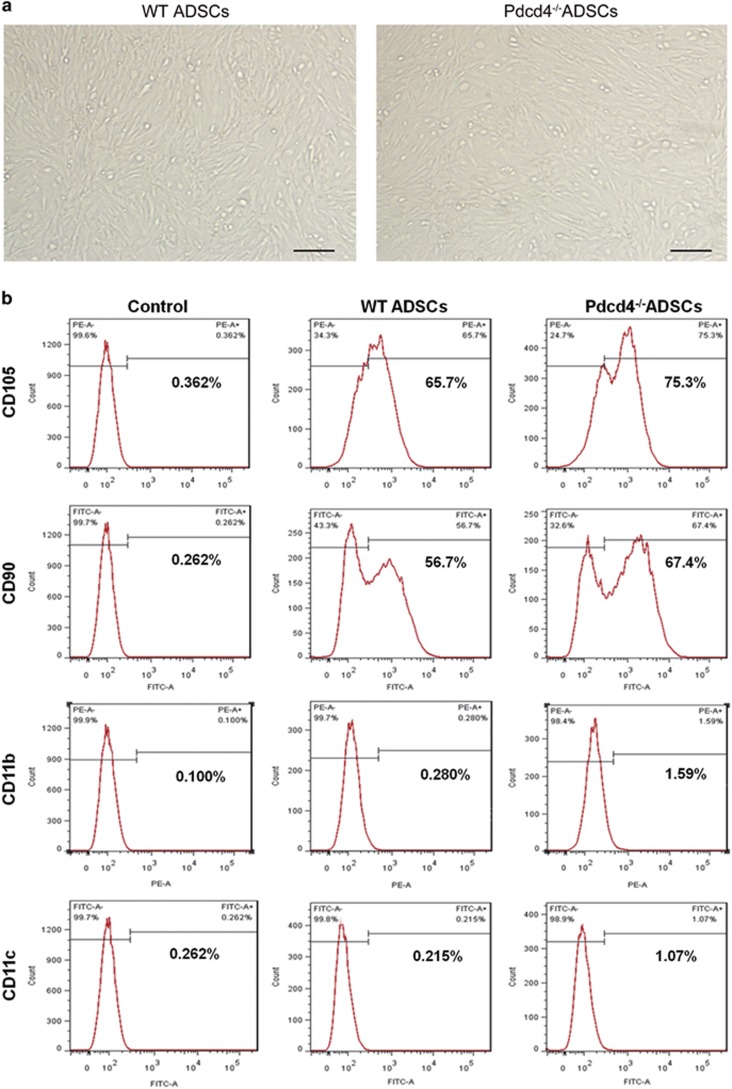
Pdcd4 deficiency increases the expression levels of stem cell-related phenotypes on ADSCs. ADSCs were isolated from epididymal fat pads of WT and Pdcd4^−/−^ mice (*n⩾*8 per group) and expanded in DMEM containing 10% FBS and bFGF (5 ng/ml). (**a**) The morphology of WT and Pdcd4^−/−^ ADSCs was examined by light microscope. Scale bar=200 *μ*m. (**b**) The expression of stem cell-related positive markers CD90 and CD105 and negative makers CD11b and CD11c was detected by flow cytometry. Data are representative of three independent experiments

ADSCs from wild-type (WT) and Pdcd4-deficient (Pdcd4^−/−^) mice fed on normal diet (ND) were isolated and expanded *in vitro*, and stem cell-related phenotypes were detected. As shown in Figure 1a, no morphological difference was observed between WT and Pdcd4^−/−^ ADSCs. Stem cell-related surface markers CD105 and CD90 were positively expressed on both WT and Pdcd4^−/−^ ADSCs, together with negative or rare expression of CD11b and CD11c. Differently, Pdcd4^−/−^ ADSCs displayed relatively higher levels of CD105 and CD90 as compared with WT ADSCs (Figure 1b), indicating that Pdcd4 deficiency possibly causes the promoting effects on ADSC stemness.

### Pdcd4 deficiency enhances the stemness of ADSCs in WAT

**Figure 2:**
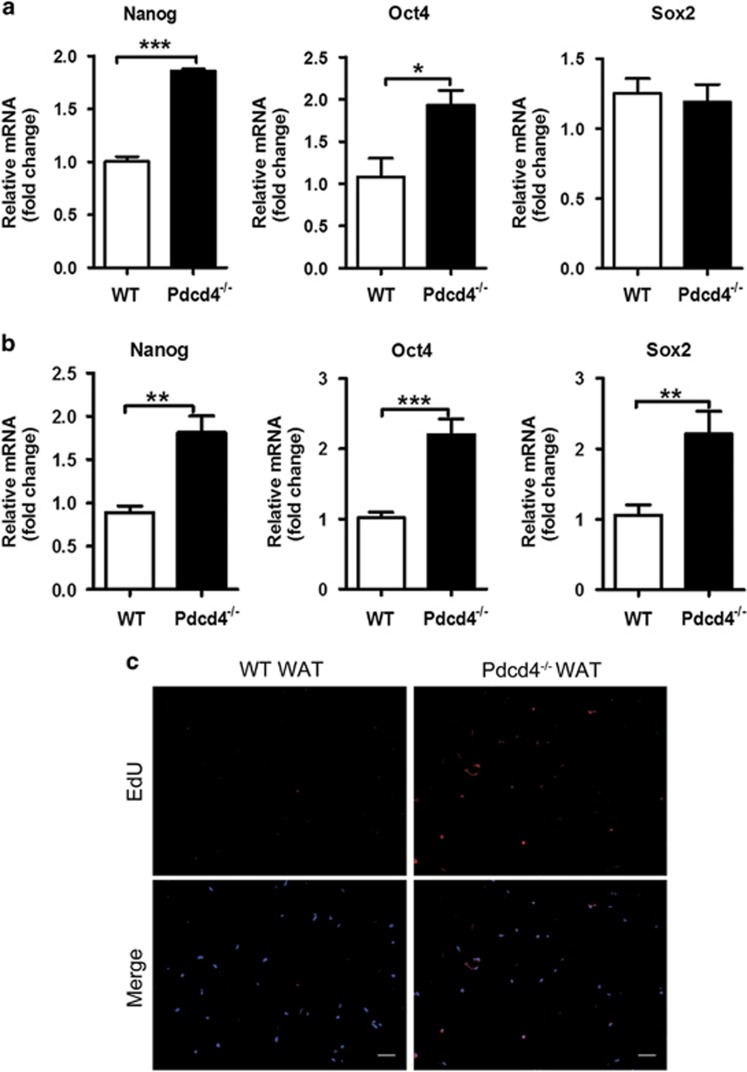
Pdcd4 deficiency increases the stemness of ADSCs in WAT. (**a**) ADSCs from WT and Pdcd4^−/−^ mice were isolated and expanded as described in Figure 1, the gene expression of stemness markers on ADSCs were measured by qPCR. Bars represent mean±S.E.M. *P<0.05, ***P<0.001. (**b**) The primary stromal vascular fraction was isolated from epididymal fat pads of WT and Pdcd4^−/−^ mice (*n*=4 per group), the gene expression of stemness markers were examined by qPCR. Bars represent mean±S.E.M. **P<0.01, ***P<0.001. (**c**) WT or Pdcd4^−/−^ mice (*n*=4) was intraperitoneally injected with 100 *μ*g EdU. After 24 h, the epididymal fat pads were collected and paraffin-embedded sections were prepared. The incorporation of EdU in dividing cells was examined by fluorescence signals and visualized with fluorescence microscope. The original magnification is 100. Scale bar=100 *μ*m

To investigate the effects of Pdcd4 on ADSC stemness, we further detected the mRNA levels of stemness markers on ADSCs. Significantly higher levels of Nanog and Oct4 mRNA, but not Sox2 mRNA, were detected on Pdcd4^−/−^ ADSCs as compared with WT ADSCs (Figure 2a). Considering that these ADSCs underwent culture and expansion *in vitro*, we next compared the mRNA levels of the stemness markers between ND-fed WT and Pdcd4^−/−^ mice, in freshly isolated stromal vascular fraction from epididymal fat pads containing primary ADSCs. Intriguingly, all the detected stemness markers including Nanog, Oct4 and Sox2 showed significantly higher levels in stromal vascular fraction from Pdcd4^−/−^ mice as compared with that from WT mice (Figure 2b), suggesting that Pdcd4 deficiency increases the stemness of ADSCs.

To further confirm the enhancement of ADSC stemness in Pdcd4^−/−^ mice, we tracked the cell-dividing efficiency in epididymal fat pads by intraperitoneal injection of 5-ethynyl-2′-deoxyuridine (EdU) into ND-fed WT and Pdcd4^−/−^ mice. After 24 h, a rare EdU signal was detected in epididymal fat tissues from WT mice, while obviously higher percentages of EdU-positive cells were observed in those from Pdcd4^−/−^ mice (Figure 2c), thus indicating that Pdcd4 deficiency could moderately accelerate the cell proliferation and maintain effective self-renewal of ADSCs in WAT.

### Pdcd4 deficiency increases the proliferation of ADSCs through promoting S-phase entry

**Figure 3:**
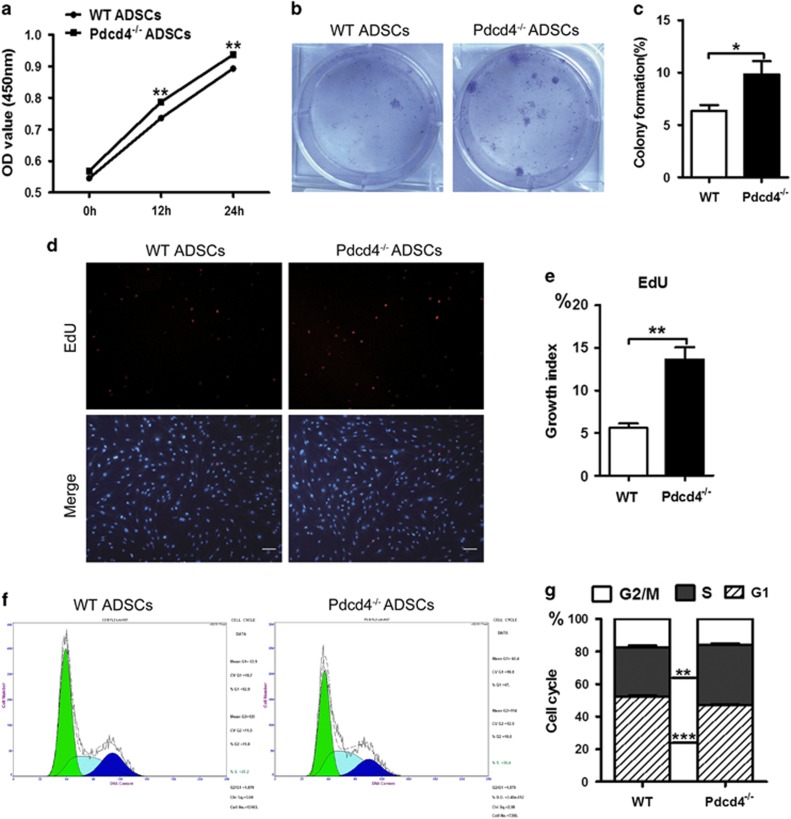
Pdcd4 deficiency increases the proliferation of ADSCs through promoting S-phase entry. (**a**) The cell proliferation of WT and Pdcd4^−/−^ ADSCs was tested using CCK-8 assay at indicated time points. Data are representative of three independent experiments. (**b**, **c**) Colony formation developed by WT and Pdcd4^−/−^ ADSCs was measured by Crystal Violet staining after 7 days' culture *in vitro*. Representative (**b**) and statistic (**c**) data are from two independent experiments. Bars represent mean±S.E.M. *P<0.05. (**d**, **e**) WT and Pdcd4^−/−^ADSCs entering S phase were determined by EdU incorporation assay. Fluorescence signals were examined by fluorescence microscope. Typical (**d**) and statistic (**e**) data are representative of three independent experiments. The original magnification is 100. Scale bar=100 *μ*m. Bars represent mean±S.E.M. **P<0.01. (**f**, **g**) Cell cycle profile of WT and Pdcd4^−/−^ADSCs was stained with propidium iodide and analyzed by flow cytometry, typical (**f**) and statistic (**g**) data are from three independent experiments. Bars represent mean±S.E.M. **P<0.01, ***P<0.001

Next, we tested the effects of Pdcd4 deficiency on the proliferation of ADSCs. The proliferation curve, as determined by Cell Counting Kit-8 (CCK-8) assay, showed that Pdcd4^−/−^ ADSCs had higher viability compared with WT ADSCs at 12 and 24 h (Figure 3a), indicating the early pro-proliferative effect of Pdcd4 deficiency on ADSCs. These results were confirmed by colony formation assays, which showed an increased amount of colonies developed by Pdcd4^−/−^ ADSCs as compared with WT ADSCs after 7 days of growth (Figures 3b and c). As EdU could incorporate into proliferative cells during DNA synthetic S phase, we further checked the cells at the S phase by EdU staining. Figures 3d and e showed that the EdU-positive cells that entered S phase obviously increased in Pdcd4^−/−^ ADSCs compared with those in WT ADSCs. Cell cycle analysis further substantiated that Pdcd4 deficiency promotes the G1/S phase transition, as suggested by higher percentages of cells at the S phase and lower percentages of cells at G1 phase in Pdcd4^−/−^ ADSCs than those in WT ADSCs (Figures 3f and g). Collectively, these data suggest that Pdcd4 deficiency increases the proliferation of ADSCs by promoting S-phase entry.

### AKT activation is responsible for the S-phase elevation caused by Pdcd4 deficiency in ADSCs

**Figure 4:**
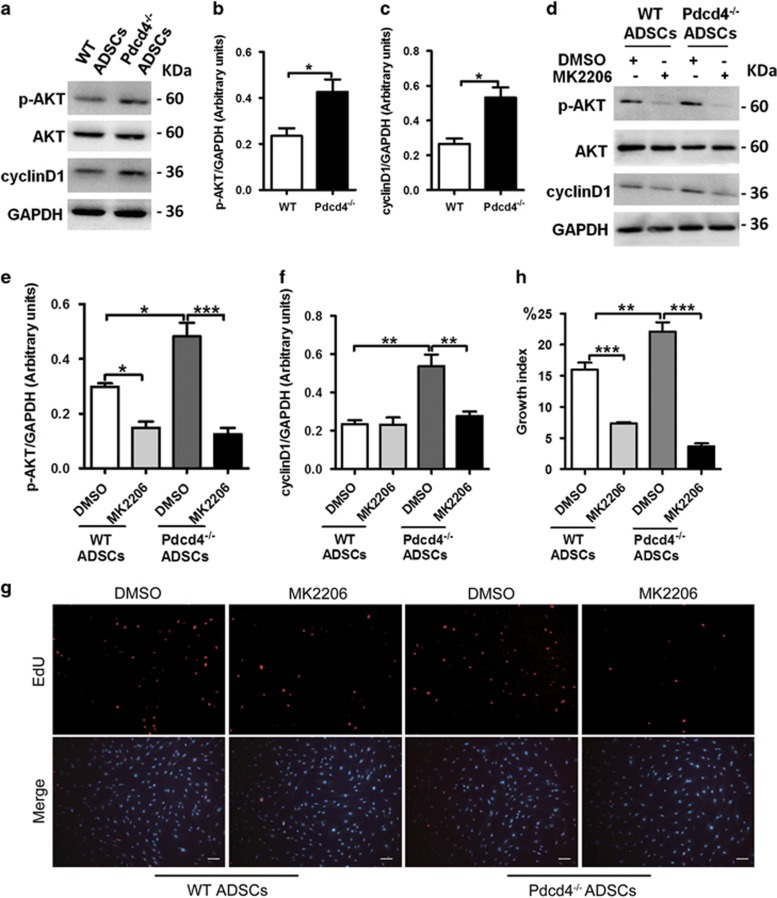
AKT activation is responsible for the S-phase elevation caused by Pdcd4 deficiency in ADSCs. WT and Pdcd4^−/−^ADSCs were cultured in the presence or absence of MK2206 (2 *μ*M) overnight, then the cells were collected for the following experiments. (**a**–**f**) Protein levels of p-AKT, AKT and cyclinD1 were detected by western blot assay. Representative (**a**, **d)** and statistical (**b**, **c**, **e**, **f**) data are shown. Bars represent mean±S.E.M. *P<0.05, **P<0.01, ***P<0.001. (**g**, **h**) ADSCs entering S phase were determined by EdU incorporation assay, fluorescence signals were examined by fluorescence microscope. The original magnification is 100. Scale bar=100 *μ*m. Typical (**g**) and statistic (**h**) data are representative of three independent experiments. Bars represent mean±S.E.M. **P<0.01, ***P<0.001

It has been well recognized that AKT signaling is necessary for the cell survival and proliferation.^34, 35^ To clarify the roles of AKT pathway in S-phase elevation of Pdcd4^−/−^ ADSCs, we detected the activation status of AKT in WT and Pdcd4^−/−^ ADSCs. Compared with WT ADSCs, Pdcd4^−/−^ ADSCs had a significant upregulation in AKT phosphorylation. Notably, enhanced AKT activation in Pdcd4^−/−^ ADSCs was accompanied by a marked increase in the expression of cyclinD1, a main regulator of G1/S phase transition,^36, 37^ which was consistent with the changes of cell cycle in Figures 3f and g (Figures 4a–c).

To confirm the contribution of AKT signaling to increased proliferation of Pdcd4^−/−^ ADSCs, inhibitor MK2206 was used to inhibit AKT activation. In WT ADSCs, blockade of AKT signaling significantly inhibited cell proliferation, but had no significant influence on the decline of cyclinD1, suggesting that AKT activation is involved in the proliferation of WT ADSCs partially independent on cyclinD1. While in Pdcd4^−/−^ ADSCs, the expression of cyclinD1 decreased substantially once AKT activation was blocked (Figures 4d–f); a sharp decrease in proliferation as evidenced by very few detectable EdU-positive cells was consistently observed in Pdcd4^−/−^ ADSCs (Figures 4g–h). These data suggest that AKT activation caused by Pdcd4 deficiency is a pivotal contributor to promote S-phase entry of ADSCs, which is dependent on cyclinD1.

### Pdcd4 deficiency drives the transdifferentiation of ADSCs into beige adipocytes

**Figure 5:**
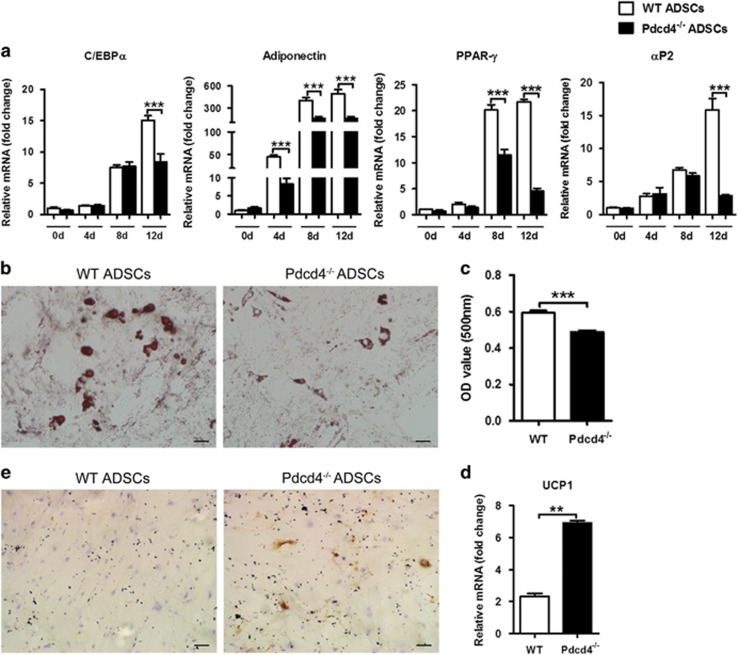
Pdcd4 deficiency drives the transdifferentiation of ADSCs from white adipocytes into beige adipocytes. Adipogenic induction was performed using white adipogenic differentiation medium for 12–18 days. (**a**) The gene expression of white adipocyte markers was measured by qPCR at day 0, 4, 8 and 12 during the induction. Bars represent mean±S.E.M. ***P<0.001. (**b**, **c**) Lipid contents were visualized using Oil Red O staining (**b**) and quantified by elution of Oil Red O (**c**). The original magnification is 200. Scale bar=50 *μ*m. Bars represent mean±S.E.M. ***P<0.001. (**d**, **e**) The expression of UCP1 was examined by qPCR (**d**) and immunocytochemistry (**e**). Original magnification is 100. Scale bar=100 *μ*m. Data are representative of three independent experiments. Bars represent mean±S.E.M. **P<0.01

To determine the role of Pdcd4 in ADSC differentiation, we further performed classical white adipogenic differentiation using WT and Pdcd4^−/−^ADSCs, respectively. As shown in Figure 5a, both WT and Pdcd4^−/−^ ADSCs underwent a rapid upregulation of white adipocyte markers including C/EBP*α*, adiponectin, PPAR-*γ* and *α*P2 during the adipogenic differentiation; whereas interestingly, the expression levels of these genes in differentiating Pdcd4^−/−^ ADSCs were obviously lower than those in differentiating WT ADSCs at 12 day post induction. For the mRNA levels of adiponectin and PPAR-*γ*, the decline occurred early at 4 or 8 days post induction. Oil Red O staining exhibited that both WT and Pdcd4^−/−^ ADSCs possessed adipogenic differentiation potency, but relatively lower levels of lipids were detected in differentiated Pdcd4^−/−^ adipocytes than those in differentiated WT adipocytes (Figures 5b and c). These data indicate that Pdcd4 deficiency could decrease the capacity of ADSCs to differentiate into white adipocytes.

Different from large and unilocular lipid droplets in differentiated WT adipocytes, much small and multilocular lipid droplets were observed in differentiated Pdcd4^−/−^ adipocytes, displaying a typical brown-like adipocyte property (Figure 5b). To clarify the possible role of Pdcd4 deficiency in the differentiation direction of ADSCs, we further checked the mRNA levels of related brown adipocyte maker UCP1 in differentiating ADSCs. As shown in Figure 5d, the mRNA levels of UCP1 were remarkably higher in differentiated Pdcd4^−/−^ adipocytes than those in WT adipocytes at 12 days post induction. The expression of UCP1 was confirmed by immunocytochemistry, which showed an apparent protein expression in differentiated Pdcd4^−/−^ adipocytes, in contrast with lesser protein expression in WT adipocytes (Figure 5e). These data revealed that Pdcd4 deficiency might, at least partially, drive the transdifferentiation of ADSCs from white adipocyte into UCP1-expressing beige adipocytes.

### Pdcd4 deficiency drives the formation of beige adipose tissues in WAT from HFD-fed mice

**Figure 6:**
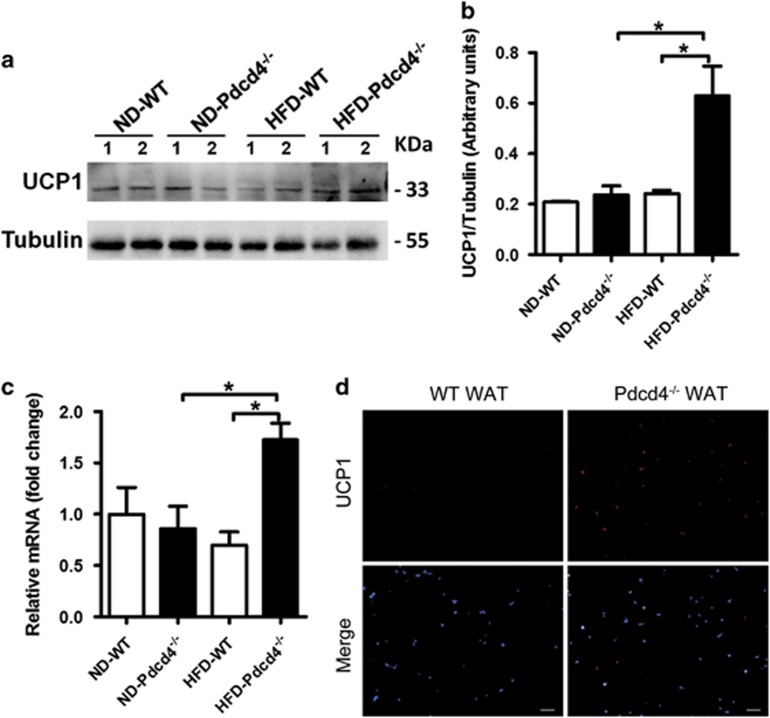
Pdcd4 deficiency induces UCP1-expressing beige adipocytes in WAT of mice in response to HFD. WT and Pdcd4^−/−^ male mice (*n*=4 per group) were fed on ND or HFD for 24 weeks, then the epididymal fat pads were collected for assay. (**a**–**c**) Protein (**a**, **b**) and mRNA (**c**) levels of UCP1 expression in WAT from indicated mice. Bars represent mean±S.E.M. *P<0.05. (**d**) *In situ* expression of UCP1 was examined by immunofluorescence staining on WAT sections from indicated mice. Original magnification is 100. Scale bar=100 *μ*m

*In vitro* studies verified that Pdcd4 deficiency could drive ADSC differentiation into UCP1-expressing beige adipocytes. To confirm whether Pdcd4 deficiency caused similar effects *in vivo*, we detected the protein and mRNA levels of UCP1 in epididymal WAT from Pdcd4^−/−^ and WT mice fed on HFD. Little amounts of UCP1 were detected in WAT from ND-fed WT and Pdcd4^−/−^ mice. In response to HFD, WAT from Pdcd4^−/−^ mice displayed an obvious elevation in UCP1 expression both on protein and mRNA levels, while WAT from WT mice showed no significant change. Compared with WT obese mice, HFD-fed Pdcd4^−/−^ mice expressed much higher levels of UCP1 on WAT (Figures 6a–c). These results were strengthened by the obvious increase in UCP1 expression accompanied by inhibited adipocyte hypertrophy on WAT sections from HFD-fed Pdcd4^−/−^ mice compared with those from WT obese mice (Figure 6d). These data were consistent with the fact that HFD-fed Pdcd4^−/−^ mice exhibit a lean phenotype with high energy consumption,^32^ demonstrating that Pdcd4 deficiency in mice promotes beige adipocyte differentiation in WAT upon HFD feeding.

### Pdcd4 deficiency increases the levels of lactate produced by ADSCs

**Figure 7:**
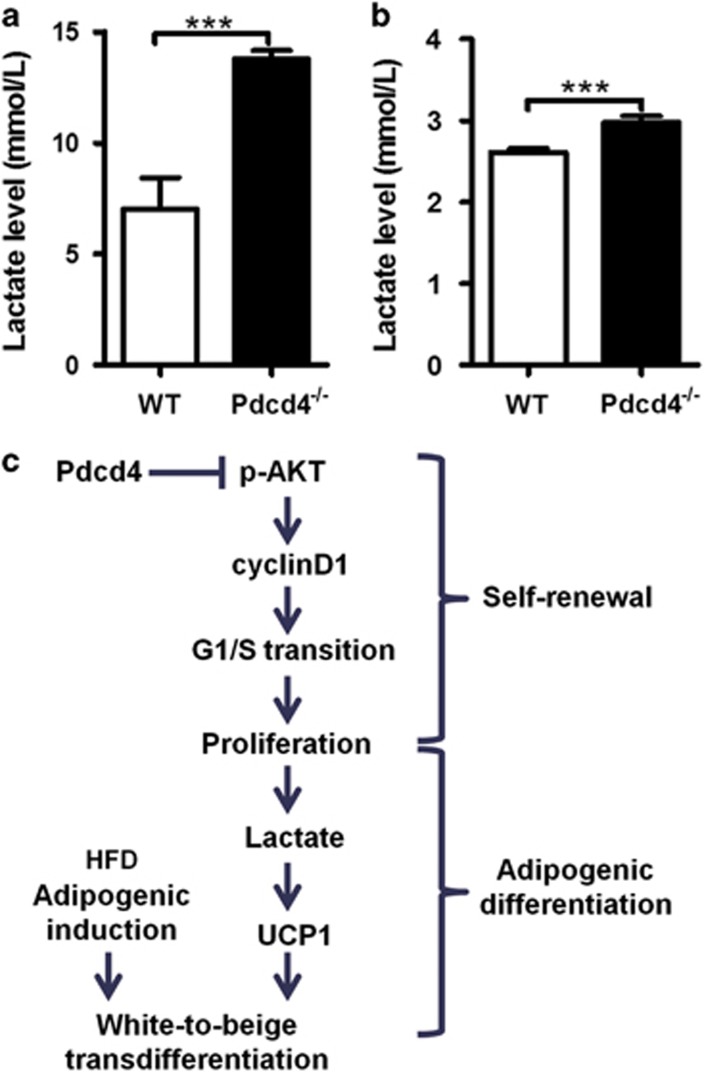
Pdcd4 deficiency increases the levels of lactate produced by ADSCs. (**a**) WT and Pdcd4^−/−^ADSCs were cultured in DMEM containing 10% FBS and bFGF (5 ng/ml) for 3 days; the supernatants (*n*=4 per group) were collected for lactate assay. (**b**) WT and Pdcd4^−/−^ADSCs were subjected to adipogenic induction under adipogenic differentiation medium. At 12 days post induction, the supernatants (*n*=4 per group) from one day of cell culture were collected for lactate detection. Bars represent mean±S.E.M. ***P<0.001. (**c**) Working model of Pdcd4 on ADSC function. Pdcd4 targeting AKT activation restrains ADSC self-renewal by suppressing G1/S transition dependent on cyclinD1. Upon adipogenic differentiation, the restriction of proliferation by Pdcd4 causes reduction in lactate levels and suppression in UCP1 expression, thus impeding the transdifferentiation of ADSCs into beige adipocytes

A very recent study has reported the direct effects of lactate on the browning of white adipocytes.^15, 38^ Considering the rapid proliferation of ADSCs caused by Pdcd4 deficiency, we speculated that more lactate might be produced by Pdcd4^−/−^ ADSCs because of an increase in anaerobic glycolysis as compared with WT ADSCs. So, we tested the lactate levels in the supernatants from WT and Pdcd4^−/−^ ADSC culture. As expected, a remarkable elevation of lactate was observed in Pdcd4^−/−^ ADSCs compared with WT ADSCs (Figure 7a). Even in differentiating Pdcd4^−/−^ ADSCs at 12 days post induction, more lactate was also detected, which echoed with the relatively higher levels of UCP1 compared with those in WT ADSCs (Figure 7b). These data linked lactate with the white-to-beige transdifferentiation of ADSCs, and revealed lactate as a pivotal contributor to the occurrence of UCP1-expressing beige adipocytes in differentiating Pdcd4^−/−^ ADSCs.

## Discussion

Adult stem cells are a keystone of tissue homeostasis by balancing self-renewal and differentiation divisions.^39^ Stemness maintenance characterized by vigorous self-renewal and differentiation potency is essential for ADSC function particularly in physiological adipogenesis. Pdcd4 has been well documented as a tumor suppressor, but the possible roles in ADSCs remain lacking. In the present study, we provide evidences that Pdcd4 serves as a critical controller that limits the stemness of ADSCs, which not only restrains the self-renewal of ADSCs, but also represses the transdifferentiation of ADSCs into beige adipocytes in response to HFD challenge.

Pdcd4 deficiency brings no observed morphological change on ADSCs, but increases the expression of stem cell-associated markers CD105 and CD90, thus promoting us to check the expression of key stemness markers Oct4, Sox2 and Nanog expressed on ADSCs.^40^ Increased levels of stemness markers were verified on *in vitro* expanded Pdcd4^−/−^ ADSCs as well as *in situ* WAT from Pdcd4^−/−^ mice, indicating that Pdcd4 may act on ADSCs and impede their stemness. Further evidences from cell proliferation, colony formation and cell cycle assays demonstrated that Pdcd4 deficiency caused a moderate acceleration in ADSC self-renewal. This observation was confirmed *in vivo* as evidenced by EdU incorporation assay showing increased efficiency of cell dividing in WAT from Pdcd4^−/−^ mice. The anti-proliferative effects of Pdcd4 on ADSCs exhibits similarity with those on tumor cells, because Pdcd4 has been recognized to inhibit cell proliferation and neoplastic transformation in various types of tumors through several different pathways.^26^ Here, we point out that Pdcd4 gets in the way of ADSCs entering the S phase by repressing AKT activation. Blocking AKT pathway in Pdcd4^−/−^ ADSCs caused marked reduction in cyclinD1 together with almost vanished S-phase entry. However, different from the protective function against tumor, Pdcd4 exerts unfavorable effects on ADSCs that contribute to obesity and associated metabolic syndromes. In response to surplus of calorie, the restriction of ADSC self-renewal by Pdcd4 may lead to shortage of ADSCs, thereby triggering pathological adipocyte hypertrophy other than physiological adipocyte hyperplasia. Therefore, Pdcd4 might be involved in the exhaustion of ADSC stemness upon long-time HFD stimulation, which contributes to the development of pathological obesity.

Previous data have shown that HFD-fed Pdcd4^−/−^ mice displayed relatively normal adipocyte morphology in WAT and obviously higher energy consumption compared with WT obese mice.^32^ Besides the explanation that Pdcd4 deficiency causes enhanced BAT efficiency, here we propose a new viewpoint that Pdcd4 deficiency mediates the transdifferentiation of ADSCs into beige adipocytes in WAT. This observation is supported by several lines of evidences. First, although classical white adipogenic induction could induce the adipogenic differentiation of both WT and Pdcd4^−/−^ ADSCs, differently, Pdcd4^−/−^ ADSCs showed significantly reduced white adipogenesis as evidenced by decreased mRNA levels of white adipocyte markers C/EBP*α*, PPAR-*γ*, adiponectin and *α*P2 during the process of differentiation. Second, differentiated Pdcd4^−/−^ adipocytes showed reduced lipid contents accompanied by small and multilocular lipid droplets, indicating the possible occurrence of new type of adipocytes different from classical white adipocytes. Third, the expression of UCP1 on both protein and mRNA levels was increased in differentiated Pdcd4^−/−^ adipocytes as compared with differentiated WT adipocytes, which was strengthened by the observation showing an increase in UCP1 expression on WAT from HFD-fed Pdcd4^−/−^ mice compared with those from WT obese mice. As a result of rapid proliferation, more lactate produced by anaerobic glycolysis might be an important contributor to UCP1 expression and white-to-beige transdifferentiation of Pdcd4^−/−^ ADSCs, whereas other possible mechanisms by which Pdcd4 regulates UCP1 expression remain to be unveiled. As UCP1 is capable of triggering a program of respiration and energy expenditure, the transdifferentiation of ADSCs into UCP1-expressing beige adipocytes might be another explanation that explicates the higher energy consumption in HFD-fed Pdcd4^−/−^ mice as well as the resistance to obesity.

Collectively, these findings reveal the novel actions of Pdcd4 on ADSC stemness (Figure 7c), thereby adding new mechanisms on the list of diet-induced obesity and proposing potential approaches to the therapy for obesity and associated diseases. A potential strategy to fight obesity is to promote white-to-beige transdifferentiation by downregulating the expression of Pdcd4 on ADSCs. However, technical knockout or knockdown of Pdcd4 gene probably brings the risk for tumorigenesis, for Pdcd4 has been well recognized as a tumor suppressor gene. Therefore, ADSC-specific knockout or knockdown of Pdcd4 gene in adipose tissues is important to prevent the undesirable effects on other cells during obesity treatment. It is also necessary to optimize the time period when regulating the expression of Pdcd4, so as to achieve a balance on energy metabolism. Furthermore, more biological processes regulating Pdcd4 expression remain to be further investigated and applied into the therapy for obesity-related pathologies.

## Materials and Methods

### Animals

Pdcd4^−/−^ mice on C57BL/6 background were kindly provided by Youhai H Chen, University of Pennsylvania School of Medicine. WT and Pdcd4^−/−^ mice at the age of 10–12 weeks were used for ADSC isolation. WT and Pdcd4^−/−^ mice at the age of 8 weeks were fed on HFD (15% w/w fat and 0.25% cholesterol) for 24 weeks to induce obesity, mice fed on ND were used as lean controls. All animal studies were approved by the Ethical Review Committee of Shandong University School of Medicine, and all experimental procedures were performed in accordance with the institutional guidelines for animal care and utilization.

### Isolation and expansion of ADSCs

Epididymal fat pads from WT and Pdcd4^−/−^ mice were minced and digested with Krebs-Ringer Bicarbonate buffer containing 2 mg/ml collagenase (Worthington, Lakewood, NJ, USA) for approximately 45 min at 37 °C with gentle agitation. After a filtration through a 100-*μ*m mesh, stromal vascular fraction was incubated in completed Dulbecco's modified Eagle's medium containing 10% fetal bovine serum (Invitrogen, Carlsbad, CA, USA) and 5 ng/ml basic fibroblast growth factor (Peprotech Group, Rocky Hill, NJ, USA). After removal of non-adherent cells, the medium was changed every 3 days. The third to fifth passages of ADSCs were used for the experiments.

### Cell proliferation assays

ADSCs from WT and Pdcd4^−/−^ mice were seeded at a density of 2500 cells per well in 96-well plates and incubated at 37 °C for indicated times. Before the end of the culture, 10 *μ*l of CCK-8 (Dojindo, Tokyo, Japan) was added for 1 h of incubation. The absorbance was measured at 450 nm to calculate the numbers of viable cells in each well. Each measurement was performed in sextuplicate. For colony formation assays, ADSCs from WT and Pdcd4^−/−^ mice were seeded in six-well plates at a density of 200 cells per well and cultured at 37 °C for 7 days. At the end of the incubation, the cells were fixed with 100% methanol and stained with 0.1% (w/v) crystal violet. Megascopic cell colonies were counted using Image-Pro Plus 6.0 software (Media Cybernetics, Bethesda, MD, USA). Each measurement was performed in triplicate.

### EdU incorporation assay

To detect the cell proliferation *in vitro*, ADSCs from WT and Pdcd4^−/−^ mice were seeded at a density of 2500 cells per well in 96-well plates. After overnight incubation at 37 °C, 10 *μ*M EdU were added for 6 h of incubation before the harvest. In some experiments, 2 *μ*M AKT inhibitor MK2206 (Selleck, Houston, TX, USA) was added during the culture. For *in situ* detection of the cell dividing of ADSCs in adipose tissues, each WT or Pdcd4^−/−^ mice was intraperitoneally injected with 100 *μ*g EdU. After 24 h, the epididymal fat pads were collected and fixed in 4% paraformaldehyde, then were prepared into 4-*μ*m paraffin-embedded sections. EdU detection was performed by Cell-Light EdU Apollo567 Cell Tracking Kit (RiboBio, Guangzhou, China) according to the manufacturer's instruction. Fluorescence signals were visualized with fluorescence microscope (Axio Imager A2, Carl Zeiss, Jena, Germany).

### Cell cycle assays

ADSCs from WT and Pdcd4^−/−^ mice were seeded in 10-mm culture dishes at a density of 2 × 10^5^ cells per dish and cultured at 37 °C overnight. After digestion with 0.25% trypsin, the cells were fixed with ice-cold 70% ethyl alcohol at 4 °C overnight, and then were incubated with propidium iodide at 4 °C for 30 min. The cells were acquired and data were analyzed with Cytomics FC500 (Beckman Coulter, Pasadena, CA, USA).

### Adipogenic differentiation of ADSCs

ADSCs from WT and Pdcd4^−/−^ mice were seeded in six-well plates at a density of 5 × 10^4^ cells/ml and cultured at 37 °C until fused completely. Adipogenic differentiation was performed using classical white adipogenic differentiation medium (Cyagen Biosciences, Santa Clara, CA, USA) according to the manufacturer's instruction. At day 18 post induction, adipogenesis was evaluated by Oil Red O staining (Sigma-Aldrich, San Francisco, CA, USA). The optical density value was measured at 500 nm after eluting Oil Red O with 100% isopropanol.

### Flow cytometry

To detect the surface markers of ADSCs, ADSCs from WT and Pdcd4^−/−^ mice were stained with fluorescein isothiocyanate-labeled antibodies (Abs) against mouse CD90 and CD11c, and phycoerythrin-labeled Abs against mouse CD105 and CD11b (eBioscience, San Diego, CA, USA), using corresponding isotypic Abs as controls. Cells were acquired using FACS Calibur Flow Cytometer (BD Biosciences, San Jose, CA, USA) and data were analyzed by Flowjo software.

### Immunocytochemistry

After 18 days of adipogenic differentiation, cells planted in 24-well chamber slides were fixed in 4% paraformaldehyde and blocked with 10% goat serum, then the cells were incubated with primary Ab against UCP1 (Proteintech Group, Rocky Hill, NJ, USA) overnight at 4 °C, followed by avidin-biotinylated HRP-conjugated secondary Ab (ZSGB-BIO, Beijing, China) for 1 h at 37 °C. Diaminobenzidine (ZSGB-BIO) was used for color reaction, and hematoxylin was used for counter-staining nuclei.

### Immunofluorescence

Adipose tissues from HFD-fed WT and Pdcd4^−/−^ mice were fixed in 4% paraformaldehyde and prepared into 4-*μ*m paraffin-embedded sections. Immunofluorescence staining was performed according to a standard protocol. Briefly, after blocking with 10% goat serum, the sections were stained with anti-UCP1 primary Ab followed by Alexa 594-congjugated secondary Ab (Proteintech Group). Cell nuclei were stained by 4, 6-diamidino-2-phenylindole (Beyotime Biotechnology, Shanghai, China). Immunofluorescence signals were visualized with fluorescence microscope.

### Lactate detection

ADSCs or differentiating ADSCs from WT and Pdcd4^−/−^ mice were cultured or induced for indicated time periods, supernatants from cell culture were collected, and extracellular lactate levels were tested by Lactic Acid LD Assay Kit (Nanjing Jiancheng Bioengineering Institute, Nanjing, China) as per the instruction.

### Quantitative PCR (qPCR)

Total RNA was extracted using RNAfast200 (Fastagen, Shanghai, China) or Trizol (TIANGEN BIOTECH, Beijing, China), and reversely transcripted with ReverTra Ace qPCR RT Kit (TOYOBO Life Science, Shanghai, China). qPCR was carried out using SYBR Green Master Mix (CWbiotech, Beijing, China). The relative mRNA levels of genes was calculated using 18 s rRNA or GAPDH as an internal control. Gene-specific primers were listed in Table 1.

### Western-blot assay

Equal amount of proteins from cell or tissue lysates was loaded on SDS-PAGE gels. After electrophoresis, proteins were transferred to PVDF membranes. The membranes were blocked with 5% bovine serum albumin for 3 h, and blotted with the indicated primary Abs against UCP1 (Proteintech Group), AKT (Epitomics, Burlingame, CA, USA), phosphor (p)-AKT, cyclinD1 (Cell Signaling Technology, Beverly, MA, USA), GAPDH and Tubulin (Proteintech Group) overnight at 4 °C, followed by incubation with HRP-conjugated secondary Abs (ZSGB-BIO) for 1 h. The signals were detected the by SuperSignal West Pico Chemiluminescent Substrate (Pierce Biotechnology, Rockford, IL, USA).

### Statistical analysis

Statistical analysis was performed with Graph Pad prism 5 software. All data are reported as mean±standard error of the mean (S.E.M.). The significant differences were evaluated using student *t* test or one-way ANOVA. *P*<0.05 was considered statistically significant.

## Figures and Tables

**Table 1:** qRT-PCR primer pairs used in this study

Nanog	Sense: CGGTGGCAGAAAAACCAGTG Anti-sense: AAGGCTTCCAGATGCGTTCA
Sox2	Sense: GGCAAGGCAGAGAAGAGAGTG Anti-sense: TCTGGCGGAGAATAGTTGGG
Oct4	Sense: TGATCCTCGAACCTGGCTA Anti-sense: CTCAGGCTGCAAAGTCTCC
C/EBP*α*	Sense: TTGAAGCACAATCGATCCATCC Anti-sense: GCACACTGCCATTGCACAAG
Adiponectin	Sense: CCTGTTCCTCTTAATCCTGCCCA Anti-sense: ATCTCCTTTCTCTCCCTTCTCTCCA
PPAR-*γ*	Sense: GGAGCCTAAGTTTGAGTTTGCTGTG Anti-sense: TGCAGCAGGTTGTCTTGGATG
*α*P2	Sense: GTGGGATGGAAAGTCGACCA Anti-sense: ATCCAGGCCTCTTCCTTTGG
UCP1	Sense: ACTGCCACACCTCCAGTCATT Anti-sense: CTTTGCCTCACTCAGGATTGG
GAPDH	Sense: GTGTTTCCTCGTCCCGTAGA Anti-sense: ATGAAGGGGTCGTTGATGGC
18S rRNA	Sense: GCCTGAGAAACGGCTACCACAT Anti-sense: CCGCTCCCAAGATCCAACTACG

## Abbreviations

ADSC: adipose-derived stem cell
Pdcd4: programmed cell death 4
HFD: high-fat diet
ND: normal-fat diet
WAT: white adipose tissue
BAT: brown adipose tissue
UCP1: uncoupling protein 1
WT: wild type
EdU: 5-Ethynyl-2'-deoxyuridine
CCK-8: Cell Counting Kit-8
DMEM: Dulbecco's modified Eagle's medium
FBS: fetal bovine serum
bFGF: basic fibroblast growth factor
Ab: antibody
